# Series 2: Invisible Threats: A Global Scoping Review of Risk Factors for Tuberculosis Infection

**DOI:** 10.3390/tropicalmed11040087

**Published:** 2026-03-24

**Authors:** Sonia Menon, Anthony D. Harries, Riitta A. Dlodlo, Gisèle Badoum, Mohammed F. Dogo, Olivia B. Mbitikon, Pranay Sinha, Yan Lin, Jyoti Jaju, Aung Naing Soe, Anisha Singh, Bharati Kalottee, Kobto G. Koura

**Affiliations:** 1 International Union Against Tuberculosis and Lung Disease, 75001 Paris, France; sonia.menon.consultant@theunion.org (S.M.); adharries@theunion.org (A.D.H.); rdlodlo@theunion.org (R.A.D.); gisele.badoum.consultant@theunion.org (G.B.); fall.dogo.consultant@theunion.org (M.F.D.); olivia.mbitikon.consultant@theunion.org (O.B.M.); psinha@bu.edu (P.S.); ylin.consultant@theunion.org (Y.L.); jyoti.jaju@theunion.org (J.J.); ansoe08@gmail.com (A.N.S.); anisha.singh@theunion.org (A.S.); bharati.kalottee@theunion.org (B.K.); 2 Epitech Research, 1160 Auderghem, Belgium; 3 Department of Clinical Research, Faculty of Infectious and Tropical Diseases, London School of Hygiene and Tropical Medicine, London WC1E 7HT, UK; 4 Unité de Formation et de Recherche en Sciences de la Santé (UFR/SDS), Université Joseph KI-ZERBO, Ouagadougou P.O. Box 7021, Burkina Faso; 5 Service de Pneumologie, Centre Hospitalier et Universitaire de Yalgado Ouédraogo (CNHU-YO), Ouagadougou P.O. Box 7022, Burkina Faso; 6 Section of Infectious Diseases, Boston University Chobanian and Avedisian School of Medicine, Boston, MA 02118, USA; 7 Boston Medical Center, Boston, MA 02118, USA; 8 Unité Mixte de Recherche MERIT (UMR261), Université Paris Cité, Institut de Recherche pour le Développement (IRD), 75006 Paris, France

**Keywords:** *Mycobacterium tuberculosis*, tuberculosis infection, latent tuberculosis infection, proximity and behavioural risk factors, environmental risk factors, host immune vulnerabilities

## Abstract

Background: Tuberculosis (TB) remains a major global health challenge, with *Mycobacterium tuberculosis* (*M. tuberculosis*) causing significant morbidity and mortality mainly in high-burden countries. Following exposure to *M. tuberculosis*, individuals may become infected, developing TB infection (TBI) through inhalation of the bacillus: this affects approximately one-fourth of the global population and serves as a critical reservoir for potential disease reactivation and transmission. The risk of being infected with *M. tuberculosis* is shaped by bacterial load of people with TB, contact patterns, environmental factors, and host susceptibility, particularly in high-risk congregate settings. Elucidating these determinants is instrumental for optimising TB prevention and control strategies. Methods: A preliminary PubMed search was conducted on 25 August 2024, using the keywords “latent tuberculosis infection,” “risk factors,” and “systematic review.” Targeted reviews were then performed in November 2024 to examine factors influencing progression from exposure to *M. tuberculosis* to TBI. Systematic reviews published between January 2000 and November 2024 were included. Results: The scoping review analysed eight systematic reviews, grouping findings into three key themes: (1) proximity and behavioural risk factors; (2) environmental risk factors; and (3) host immune vulnerabilities. Close contact with people with TB in crowded settings, such as dormitories, healthcare facilities, and prisons, was strongly associated with an elevated risk of TBI. Healthcare workers travelling from low- to high-incidence regions faced the highest risk due to frequent exposure to *M. tuberculosis*, while military personnel and general travellers had lower risks. Environmental exposures, including second-hand smoke and inadequate ventilation, further heightened susceptibility among children and adults. Host immune risk factors, such as advanced age, low body mass index, lack of BCG vaccination, and metabolic disorders such as diabetes, markedly increase susceptibility to TBI. The interplay between proximity, behavioural and environmental risk factors, and host immune vulnerabilities highlights the multifactorial nature of TBI risk. Conclusion: Effective TBI control demands a multifaceted approach, combining robust infection prevention and control measures, comorbidity management, and mitigation of behavioural risk factors like smoking. Tailored strategies are crucial for high-risk settings such as healthcare facilities and prisons. Multisectoral collaboration is essential to address key risk factors and protect vulnerable populations from progressing to TBI.

## 1. Introduction

Tuberculosis (TB) remains one of the most significant global public health challenges, driven by the airborne transmission of *Mycobacterium tuberculosis* (*M. tuberculosis*). According to the World Health Organization (WHO), an estimated 10.7 million people developed TB worldwide in 2024, and approximately 1.23 million people died from the disease [1]. TB continues to rank among the leading causes of death worldwide and was listed among the top 15 causes of death globally in 2021 [1]. Despite advances in diagnostic and therapeutic strategies [2], its control remains hampered by the emergence of programmatically incurable TB, which threatens to destabilise control efforts [3].

TB infection (TBI) typically progresses through three phases. Following exposure to *M. tuberculosis* through inhalation of infectious aerosols, not all exposed individuals will become infected (previously referred to as latent TB infection (LTBI)): this refers to a condition where an individual has been infected with *M. tuberculosis* but does not exhibit any symptoms or signs of TB disease. Only a subset of individuals (5–15% of people) will progress to TB disease [4]. Although rare, TBI may also occur via the digestive route, particularly through the consumption of unpasteurised milk contaminated with Mycobacterium bovis. Currently, the diagnosis of TBI is based on reactive tuberculin skin testing (TST) and/or a positive interferon-gamma release assay (IGRA) [5], with the latter being more sensitive [6]. Globally, the pooled prevalence of TBI, as estimated using IGRA, was approximately one-fourth of the population [7].

The risk of acquiring TBI is driven by a complex interplay of factors, including the characteristics of the *M. tuberculosis* strain, such as transmissibility [8], contact dynamics, environmental conditions, and host susceptibility [9]. A critical determinant is the bacterial load of the infectious individual, as higher bacillary loads result in greater release of *M. tuberculosis*-containing aerosols [10], especially during frequent or forceful coughing episodes [11]. Close and prolonged contact with an infectious source further amplifies this risk [11], particularly in high-risk environments such as households, healthcare facilities, dormitories, and prisons [12] where overcrowding, poor ventilation, and population density converge to facilitate *M. tuberculosis* transmission. Furthermore, air pollution stemming from second-hand smoke, indoor motorcycle emissions, and cooking, may further exacerbate TBI risk in child household contacts of people with TB [13], by impairing host respiratory immune protection [14]. The effects may be particularly detrimental in immunocompromised individuals, who are inherently more vulnerable to infection.

This scoping review aims to synthesise evidence on established and emerging determinants influencing progression from exposure to *M. tuberculosis* to TBI, focusing on proximity and behavioural risk factors, environmental risk factors, and host vulnerabilities from the year 2000 onward. By placing findings within the context of demographic and epidemiological transitions, this review seeks to inform targeted prevention and control strategies for diverse high-risk populations. This review constitutes the second paper in a structured series examining the natural history of tuberculosis.

## 2. Materials and Methods

### 2.1. Search Strategy and PEOS Questions

A preliminary literature search was started on 25 August 2024 to identify systematic reviews examining risk factors associated with TBI or LTBI. The search was performed using the PUBMED database using the following search string: (“latent tuberculosis infection” OR “TB infection” OR “tuberculosis infection”) AND (“risk factors”) AND (“systematic review” OR “meta-analysis”). Systematic reviews published between January 2000 and November 2024 were included to align with the objective of updating evidence and identifying emerging evidence. Based on the identified risk factors, a targeted review of systematic reviews was conducted in November 2024 to examine factors influencing progression from exposure to *M. tuberculosis* to TBI. We registered our protocol with OSF https://osf.io/gn74s/overview (accessed on 22 December 2024).

The primary research question guiding this scoping review was: What are the current risk factors for TBI? To structure the review, the following PEOS question was formulated to systematically scope relevant literature.

*Population:* Individuals of all ages and risk groups at risk of TBI.
*Exposure:* Any risk factors for TBI
*Outcome:* TBI
*Study Design:* Systematic reviews published within the specified timeframe (2000–2024)

### 2.2. Inclusion and Exclusion Criteria

Systematic reviews published from 2000 that met the eligibility criteria were screened based on their titles and abstracts, followed by a full-text review to confirm relevance. Original research, commentaries, editorials, and other types of literature, and systematic reviews that only included studies before 2000 were excluded.

### 2.3. Data Extraction, Synthesis, and Reporting

Data from the included studies were independently extracted by two reviewers using a predefined extraction framework (Study population and setting, Number of included studies, Identified risk factors, Effect measures reported, Quality assessment methods used) to ensure reliability and minimise bias. Key findings were synthesised into categories that emerged organically from the data based on the reviewed literature. The scoping review adhered to PRISMA-ScR (Preferred Reporting Items for Systematic Reviews and Meta-Analyses, Extension for Scoping Reviews) guidelines [15]. The PRISMA-ScR checklist is available in the Supplementary Materials Table S1.

### 2.4. Use of Non-Stigmatising Language

We have adopted non-stigmatising and person-centred terminology throughout this manuscript when referring to TB and individuals affected by it. Terms such as “people with TB” have been used instead of “TB patients” or “TB cases”. We also use “TB infection” instead of “latent TB infection” and refer to “TB disease” rather than “active TB,” except where legacy terms are needed for clarity in cited literature. This linguistic approach aims to reduce stigma, promote respect and dignity, and reflect the evolving norms in global TB research and practice [16].

## 3. Results

### 3.1. PRISMA Flow Diagram and Systematic Review Characteristics

**Figure 1:**
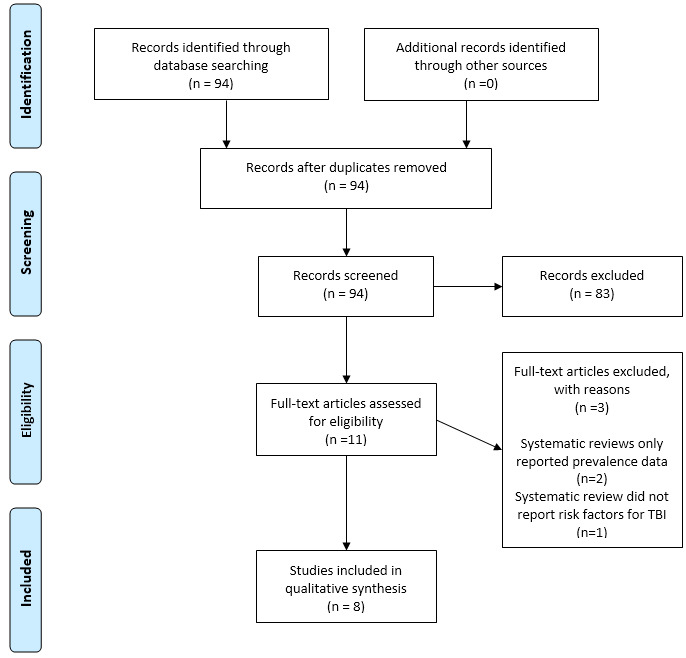
PRISMA flow diagram.

A total of 94 systematic reviews were identified in the initial search. Of these, 11 full-text reviews were assessed for eligibility. Two were excluded for reporting only on the prevalence of TBI across different groups, and one was excluded for focusing solely on risk factors for TB disease rather than TBI. Ultimately, eight systematic reviews met the inclusion criteria and were included in this scoping review. These were categorised into three broad themes: proximity and behavioural risk factors, environmental risk factors, and host immune vulnerabilities (see Figure 1 for the PRISMA flow diagram and Table 1 for the Systematic Review Characteristics).

### 3.2. Proximity and Behavioural Risk Factors

Progression to TBI is influenced by factors such as bacterial load in sputum, coughing intensity, and proximity to infectious individuals (WHO 1998 [9]), particularly in settings with close contact, such as student dormitories, healthcare facilities, and other crowded environments. Proximity and behavioural risk factors were examined in six systematic reviews. Yuan et al. (2022) [17] conducted a systematic review and meta-analysis examining TBI risk factors among college students. Across 50 studies from 18 countries, they found that contact with people with TB was strongly associated with TBI risk (Standardised mean difference (SMD): 1.34, 95% confidence interval (CI: 1.11–1.62)), highlighting the impact of proximity in high-density environments, such as dormitories. Clinical training, which involves repeated exposure to potentially infectious individuals, was identified as an even stronger risk factor (SMD: 1.93, 95% CI: 1.65–2.26). Lee et al. (2022) [18] reported a TBI rate of 29.94% (unknown CI) among healthcare workers (HCWs), notably higher than the global TBI prevalence of 23.0% (95% CI: 20.4–26.4%) in 2014. Within the review, two studies were cited. The first study identified prolonged patient contact, especially among nursing professionals, as a key risk factor, reporting a significantly higher infection rate among those with longer working durations (>7 days: 12/12 (100%) vs. ≤7 days: 18/43 (41.9%); *p* = 0.0002) [25]. The second study found that direct contact with the patient was associated with increased risk (OR: 2.83; 95% CI: 1.47–5.45) [26]. Kawatsu et al. (2016) [19] reviewed TBI prevalence and incidence in correctional settings, finding significantly higher rates compared to the general population. In middle- and high-burden countries, TBI prevalence among prisoners was 73.0%, compared to 40.3% in low-burden countries. Key risk factors included the duration of incarceration and history of previous incarceration, emphasising the transmission risk posed by close and repeated contact in overcrowded and poorly ventilated prison environments.

Diefenback-Eslitob et al. (2021) [20] examined TBI risk among travellers from low- to high-incidence (>100 TB cases/100,000 population) countries. Among individuals travelling for a mean/median of up to 6 months, HCWs had the highest cumulative incidence of TBI (4.3%), whereas the risk was lower for military (2.5%) and general travellers/volunteers (1.6%). Meta-regression showed no significant association between TBI incidence and travel duration or TB incidence in the destination country, highlighting the role of occupational proximity in TB transmission. Freeman et al. (2010) [21] similarly explored TBI risks among long-term travellers from low to high-incidence areas. When stratified by military and civilian studies, the cumulative risk estimate was 2.0% (99% CI: 1.6–2.4%) for military personnel, consistent with rates seen in local populations in many lower-income settings, and 2.3% (99% CI: 2.1–2.5%) for studies involving civils, reflecting increased transmission risk due to extended exposure in high-burden environments.

A systematic review and meta-analysis conducted by Meregildo-Rodriguez et al. (2024) [22] assessed the prevalence of TBI among HCWs in the Latin American and Caribbean region. The study included a pooled sample of over 15,000 HCWs, revealing a high overall prevalence of TBI, 40.98% (95% CI 34.77–47.33%) when combining both interferon-gamma release assay and TST. Risk factors for TBI included smoking, longer employment duration, direct exposure to people with TB, and specific professional roles such as nurses and physicians.

### 3.3. Environmental Risk Factors

Environmental factors play a critical role in the transmission and progression of TB, with second-hand smoke emerging as a significant contributor to TBI. Two systematic reviews have explored these associations, focusing on the impact of environmental exposures on TB risk among HCWs, as well as across different age groups and indoor environments.

The same systematic review and meta-analysis by Meregildo-Rodriguez et al. (2024) [22] identified inadequate infection prevention and control measures as significant contributors to TBI risk among healthcare workers in the Latin American and Caribbean region. Specific factors included irregular use of N95 masks, poor ventilation, and insufficient adherence to TB control guidelines. Additionally, work in high-risk environments such as emergency and radiology departments further exacerbated exposure risk. Environmental risk factors have been investigated in a systematic review, focusing on the impact of second-hand smoking and indoor air pollution on TBI. Patra et al. (2015) [23] found that exposure increased the risk of TBI (RR 1.67, 95% CI 1.12–2.48) in children and adults living in crowded conditions.

### 3.4. Host Immune Vulnerability

Host immune risk factors have been evaluated in six systematic reviews, which underscore their critical role in determining susceptibility to TBI and TB disease. Conditions impairing immune function significantly increase the risk of TBI, particularly in individuals with chronic diseases or immune-compromised states. Yuan et al. (2022) [17] identified several host immune-related factors contributing to increased TBI prevalence among college students, including older age (SMD: 1.67, 95% CI: 1.31–2.13), lack of BCG vaccination (SMD: 1.51, 95% CI: 1.06–2.16), and overweight/obesity (SMD: 1.17, 95% CI: 1.06–1.30). While environmental factors were also observed, the immune implications of these host-specific variables emphasise the need for tailored interventions targeting at-risk student populations.

Zhou et al. (2023) conducted a meta-analysis confirming that diabetes mellitus (DM) significantly increases the risk of TBI, with a pooled adjusted odds ratio (aOR) of 1.21 (95% CI: 1.14–1.29) [24]. The risk was even higher when DM was diagnosed using HbA1c (aOR: 1.56; 95% CI: 1.24–1.96), indicating that metabolic dysregulation plays a crucial role in compromising immune responses and increasing susceptibility to TBI. In the above-mentioned systematic review and meta-analysis conducted by Meregildo-Rodriguez et al. (2024) [22], in the Latin American and Caribbean region, a high overall prevalence of TBI of 40.98% among HCWs was observed, where older age was observed to be among the risk factors.

## 4. Discussion

### 4.1. Summary of Results

This review synthesises evidence on TBI risk factors from eight systematic reviews, updating and expanding the knowledge base with studies published since 2000. Systematic reviews examined proximity and behavioural risk factors, environmental factors, and host immune vulnerabilities, providing a clearer understanding of how these risks interact to drive *M. tuberculosis* transmission and progression from exposure to TBI. Close contact with TB-infected individuals, particularly in crowded settings such as healthcare facilities, student dormitories, and prisons, emerged as a constant risk factor for progression to TBI. Among travellers from low- to high-incidence countries, HCWs were at particularly high risk due to frequent exposure to people with TB, while military personnel and general travellers faced lower but still measurable risks. These findings highlight the occupational and contextual factors that influence TB transmission during travel. Moreover, environmental exposures, including second-hand smoke and indoor air pollution, were shown to increase TBI, particularly in vulnerable populations like children. Finally, immune-related factors, such as diabetes, smoking, and malnutrition, significantly increased susceptibility, highlighting the critical role of impaired immune defences.

Importantly, the transition from exposure to *M. tuberculosis* to established TBI is not uniform and reflects a complex interaction between bacillary load, exposure intensity, and host immune competence. Metabolic disorders such as diabetes may impair macrophage function and adaptive immune responses, thereby reducing the capacity to contain inhaled bacilli. Similarly, malnutrition and micronutrient deficiencies may compromise cell-mediated immunity, increasing vulnerability to infection. Environmental exposures, including second-hand smoke and indoor air pollution, can disrupt mucosal barriers and alter pulmonary immune defences, facilitating bacillary establishment following exposure. These mechanistic pathways remain incompletely understood, particularly in high-density settings where cumulative exposure interacts with host susceptibility. Future research integrating immunological profiling, exposure quantification, and longitudinal cohort designs is needed to disentangle the relative contribution of exposure intensity versus host susceptibility in determining TBI risk.

### 4.2. Public Health Impact

The public health impact of these findings is far-reaching, as they shed light on the complex, multifaceted risk factors contributing to TBI. The identification of proximity and behavioural risk factors, such as close contact with people with TB in crowded settings like healthcare facilities, schools, and prisons, and during travel to high-incidence regions, highlights the need for targeted interventions to mitigate TBI risk in high-exposure environments. These include, among others, a focus on early identification, isolation, and treatment of those with TB, infrastructure modifications (enlarged windows and open skylights) to ensure natural ventilation and airflow, avoidance of patient congestion in in-patient and out-patient departments, and personal protective measures for health workers or other high-risk settings such as correctional facilities [27,28]. These efforts should be accompanied by enhanced screening and preventive therapy for high-risk groups, alongside addressing behavioural and social factors such as malnutrition, tobacco use, and overcrowding. Expanding BCG vaccination coverage may further reduce the risk of TB, TBI, and its progression to TB disease, thereby contributing to the interruption of TB transmission in vulnerable populations. In addition, the development and deployment of new TB vaccines could play a critical role in strengthening these efforts.

Immune-compromising conditions, such as younger age, older age, diabetes, and overweight or obesity, may increase susceptibility to TBI, particularly following close exposure to people with TB. While current tests do not determine the timing of infection, identifying recent exposures or new conversions remains key to targeting TB preventive treatment where it is most effective. These individuals may also be at heightened risk from environmental factors such as second-hand smoke and indoor air pollution, which can exacerbate their susceptibility. This underscores the urgent need for policy interventions to reduce exposure in households, workplaces, and community spaces, particularly for those with compromised immunity.

### 4.3. Research Gaps

Despite the limitations, this review has identified some key clinical and epidemiological research gaps in understanding and addressing TBI risk factors. These are highlighted below.

#### 4.3.1. Behavioural Risk Factors in High-Density Settings

While smoking is a well-established risk factor for TBI, the influence of vaping remains poorly understood, along with drug use. With the rising prevalence of e-cigarette use, particularly in LMICs, it is imperative to explore how vaping interacts with smoking and other behaviours, including high alcohol consumption in high-density settings, to exacerbate TBI risk. Mathematical modelling could provide a deeper understanding of the synergistic effects of smoking, vaping, drug use, and environmental exposures on TBI susceptibility, particularly in crowded environments where close contact facilitates transmission.

Importantly, exposure to *M. tuberculosis* does not invariably lead to established TB infection. A proportion of individuals exposed to infectious aerosols appear to resist infection or clear the bacilli before measurable immune conversion occurs. While this review focused on identifying risk factors associated with TB infection, understanding protective determinants represents an equally important research frontier. Host immune resilience, genetic susceptibility, prior immune priming, microbiome composition, and environmental modifiers may contribute to resistance following exposure. However, these protective mechanisms remain incompletely characterised, particularly in high-burden and high-density settings. Future research integrating immunological profiling, longitudinal cohort designs, and exposure quantification is needed to better distinguish susceptibility factors from protective pathways. Such knowledge could improve risk stratification and inform more efficient allocation of TB preventive interventions.

#### 4.3.2. Immune-Suppressing Conditions and TBI

The rising cancer burden in LMICs, driven by demographic transitions such as population growth and ageing, underscores the need to examine the association between cancers and TBI risk. While age-standardised cancer incidence and mortality rates are declining in high-income countries, they are increasing in LMICs, particularly for haematological malignancies like leukaemia and non-Hodgkin lymphoma [29]. Malnutrition, a prevalent comorbidity in LMICs, further exacerbates immune suppression [30], which, as a corollary, may increase susceptibility to TBI. However, evidence on the optimal effectiveness and duration of targeted TB preventive treatment for immunocompromised groups, such as those with cancer or malnutrition, remains limited. Future research should prioritise propensity-matched longitudinal cohort studies, followed by systematic reviews, to better understand how different immune-suppressing conditions interact with different behavioural risk factors. These studies are essential to inform and optimise targeted preventive treatment strategies, particularly in resource-limited, high-density settings where TB and malnutrition frequently coexist.

#### 4.3.3. Vitamin D and TBI

Despite Cao et al. (2022) [31] finding no significant association between vitamin D levels and TBI, their meta-analysis revealed considerable imprecision in the results (OR = 0.51, 95% CI: 0.05–5.65), highlighting the need for more robust studies. Further well-powered research with clearer population definitions is essential to determine whether vitamin D supplementation could effectively reduce TBI risk.

#### 4.3.4. Domestic Solid Fuel Use and TBI

Although some evidence suggests an association between domestic solid fuel use and TBI, the evidence remains weak and highly heterogeneous, largely due to the low quality of the evidence currently available [32]. High-quality, large-scale studies and mathematical modelling studies are urgently needed to better assess the relationship between solid fuel use and TBI, and to estimate the magnitude of this environmental risk factor to guide public health interventions.

#### 4.3.5. Air Pollution

A recent population-based multicentric cohort study in China reported no significant association between particulate matter, PM2.5 exposure and TBI risk in older adults [14]. Nonetheless, further robust research is needed to clarify this relationship, with a particular emphasis on elucidating how air pollution interacts with other risk factors, such as malnutrition and smoking, to increase susceptibility to TBI.

### 4.4. Strengths and Limitations

Our scoping review has key strengths, a broad keyword search capturing multiple dimensions of TB risk, and no language restrictions, reducing bias and including evidence from non-English sources. However, the interpretation of data should consider certain limitations. This scoping review exclusively focused on systematic reviews, which may limit the depth of understanding of emerging TBI risk factors. By relying on systematic reviews, the analysis may have overlooked data from recent primary studies and emerging evidence not yet incorporated into existing reviews. The variability in study quality across systematic reviews may affect the reliability of the synthesised findings. Moreover, not all reviews have assessed the quality of included studies or provided a detailed description of the methods used for quality assessment. Also, the exclusion of reviews that included studies published before 2000 could have resulted in an incomplete evaluation of long-standing or historical risk factors for TBI. Finally, this scoping review relied on PubMed as the primary database, which may have resulted in the omission of relevant systematic reviews indexed exclusively in other databases, particularly those covering environmental or social science literature.

## 5. Conclusions

The progression to TBI is determined by a complex interplay of environmental, behavioural, and immune-related factors. High-risk settings such as healthcare facilities, dormitories, and prisons, coupled with prolonged contact with people with infectious TB disease, who may either be symptomatic or asymptomatic, remain key drivers of transmission. Smoking and exposure to second-hand smoke further exacerbate susceptibility, particularly in crowded environments.

Immunocompromising conditions, such as diabetes, older age, and obesity/overweight, significantly heighten the risk of TBI, underscoring the need for targeted interventions. Addressing these challenges requires a coordinated approach involving public health agencies, legislators, environmental health bodies, urban planning authorities, and researchers. Multisectoral cooperation is crucial for preventing transmission, reducing exposure in high-risk settings, and tailoring interventions for vulnerable populations. To this end, TB prevention efforts must be integrated into broader healthcare strategies that address comorbidities and high-risk behaviours to reduce the risk of TBI.

## Figures and Tables

**Table 1:** Systematic review characteristics included in the current scoping review.

Author of Systematic Review	Date Range of Eligible Studies	Number of Studies	Objective	Methodology Assessment	Conclusion
Yuan et al. (2022) [17]	Until 2022	50 studies	Estimate the pooled prevalence of LTBI and identify its risk factors.	Cochrane collaboration for analytical studies and STROBE; the quality scores of 10 studies were low	The prevalence of TBI among college students was 20% (95% CI: 17–23%) by TST and 9% (95% CI: 7–11%) by IGRA. Significant risk factors included older age, lack of BCG vaccination, contact with TB cases, clinical training, and overweight/obesity. Prevalence varies by region. Infection control measures are recommended for college students with LTBI.
Lee et al. (2022) [18]	August 2010–Dec 2018	61 studies	Understand the status and risk factors of TBI among health workers	Not available	In 2014, the global prevalence of latent TBI was estimated at 23.0% (95% uncertainty interval: 20.4–26.4%). Among healthcare workers, risk factors for TB include older age, longer duration of employment, being a nursing professional, lack of BCG vaccination, and low BMI. Healthcare workers are at increased risk of TBI, which can lead to secondary transmission to patients or colleagues. Implementing effective prevention strategies is essential to ensure early diagnosis and prompt treatment.
Kawatsu et al. (2016) [19]	Cannot access full article (Japanese)	12 studies	Report on the prevalence and risk factors for LTBI.	Full article was not accessible. Authors have been contacted	The prevalence of TBI among college students was 20% (95% CI: 17–23%) by TST and 9% (95% CI: 7–11%) by IGRA. Significant risk factors included older age, lack of BCG vaccination, contact with TB cases, clinical training, and overweight/obesity. Infection control measures are recommended for college students with TBI.
Diefenbach-Elstob et al. (2021) [20]	1994–2013	10 studies	Estimate incident LTBI and active TB among individuals travelling from low to higher TB incidence countries	2 studies were of moderate risk of bias and 1 of high risk of bias	A total of 1,154,673 travellers were observed between 1994 and 2013. The overall LTBI incidence was 2.3%, with HCWs at the highest risk (4.3%), followed by military (2.5%) and general travellers (1.6%). No significant differences in LTBI risk were found based on travel duration or destination TB burden.
Freeman et al. (2010) [21]	1995–2007	9 studies	Determine the risk for TST conversion, used as a surrogate for LTBI, in long-term travellers from low- to high-risk countries	Not available	The cumulative TBI risk, measured by TST conversion, was 2.0% (99% CI: 1.6–2.4%), with significant heterogeneity (*p* < 0.0001). Risk was 2.0% (99% CI: 1.6–2.4%) for military and 2.3% (99% CI: 2.1–2.5%) for civilians. TST conversion may overestimate LTBI risk in low-prevalence populations like travellers due to its low positive predictive value. A targeted testing strategy for long-term travellers is recommended, with further studies needed to identify high-risk groups and locations.
Mergildo-Rodriguez et al. (2024) [22]	2000–2020	38 studies	Estimate the prevalence of LTBI among HCWs and by specific role across Latin American and the Caribbean	Certainty of evidence was very low	Analysis of 15,236 HCWs, 6728 LTBI cases) across 7 LAC countries found a pooled LTBI prevalence of 34.5% (95% CI: 25.4–44.1%) for IGRA and 43.0% (95% CI: 35.5–50.7%) for TST. Overall prevalence using both tests was 40.98% (95% CI: 34.77–47.33%). LTBI was linked to longer employment, patient exposure, older age, nurses, physicians, smoking, and poor infection control.
Patra et al. (2015) [23]	1996–2014	18 studies	Explore the role of SHS exposure as a risk factor for TB among children and adults.	Newcastle-Ottawa Scale (score range: 3–9)	Children exposed to second-hand smoke exposure had a significantly higher risk of TBI (RR 1.64, 95% CI 1.00–2.83). Associations for TBI remained significant after adjustment for age, fuel use, and presence of a TB patient in the household. There was a loss of association with after adjustment for socioeconomic status and study quality.
Zhou et al. (2023) [24]	2006–2021	22 studies	Explore the association between DM and LTBI and provide essential reference for future research.	6 studies have a high risk of bias using the Cochrane Rob2 tool	The meta-analysis consisting of 68,256 participants found that DM increases TBI risk. Three cohort studies were eligible, with a pooled aRR of 1.26 (95% CI: 0.71–2.23). 19 cross-sectional studies were eligible, with a pooled aOR of 1.21 (95% CI: 1.14–1.29). In the diagnosis of DM, the pooled aOR of the HbA1c group was higher than that of self-reported group (pooled aOR: 1.56, 95% CI: 1.24–1.96 vs. 1.17, 95% CI: 1.06–1.28).

## Data Availability

The data that support the findings of the study are available from one of the first authors (K.G.K.) upon reasonable request.

## Supplementary Materials

The following supporting information can be downloaded at: https://www.mdpi.com/article/10.3390/tropicalmed11040087/s1, Table S1: Preferred Reporting Items for Systematic reviews and Meta-Analyses extension for Scoping Reviews (PRISMA-ScR) Checklist—Risk Factors for Tuberculosis Infection.
